# Appropriate dose of regorafenib based on body weight of colorectal cancer patients: a retrospective cohort study

**DOI:** 10.1186/s12885-023-11720-6

**Published:** 2023-12-21

**Authors:** Masayuki Nakashima, Kan Li, Qichen Chen, Sajith de Silva, Hal Li, Koji Kawakami, Qingyi Wei, Sheng Luo, Hong Zhao

**Affiliations:** 1 Department of Pharmacoepidemiology, Graduate School of Medicine and Public Health, Kyoto University, Kyoto, Japan; 2 MRL, Merck & Co., Inc, North Wales, PA USA; 3 Department of Hepatobiliary Surgery, National Cancer Center/National Clinical Research Center for Cancer/Cancer Hospital, Chinese Academy of Medical Sciences and Peking Union Medical College, Beijing, China; 4 Duke Cancer Institute, Duke University Medical Center, Durham, NC USA; 5 Department of Medicine, Duke University School of Medicine, Durham, NC USA; 6 Department of Population Health Sciences, Duke University, Durham, NC USA; 7 Department of Biostatistics and Bioinformatics, Duke University, Durham, NC USA

**Keywords:** Regorafenib, Reduced dose, Colorectal cancer, Cohort study, Drug therapy

## Abstract

**Purpose:**

Previous randomized studies have shown a survival benefit of using regorafenib but a high rate of adverse events in unresectable colorectal cancer patients. To reduce these adverse events and improve the tolerability, we examined the appropriate dose of regorafenib based on body weight.

**Methods:**

We used a nationwide claims database in Japan and examined the efficacy and safety of regorafenib for patients with metastatic colorectal cancer between groups divided by body weight (60 kg) and median average dose (120 mg) between 2013 and 2018. We also assessed overall survival (OS) and adverse events between these groups.

**Results:**

We identified 2530 Japanese patients (heavy weight/high dose: 513, light weight/low dose: 921, heavy weight/low dose: 452, and light weight/high dose: 644). There was no significant difference in the adverse events and OS after inverse probability treatment weighting (IPTW) adjustment between heavy weight/high dose group and light weight/low dose group (hazard ratio, HR=0.97). Among the light-weight patients, higher average dose was associated with shorter OS (IPTW adjusted HR=1.21, 95% CI 1.05 – 1.39, Table 3) while among the heavy-weight patients, there was no significant difference in OS between high and low dose groups (IPTW adjusted HR=1.14, 95% CI 0.95 – 1.37).

**Conclusion:**

The findings suggest that a low dose of regorafenib for light-weight patients may be as safe and effective as high doses for heavy-weight patients. Further studies should be conducted to identify an appropriate dose based on each patient's physique and condition.

**Supplementary Information:**

The online version contains supplementary material available at 10.1186/s12885-023-11720-6.

## Introduction

Regorafenib is an oral multi-kinase inhibitor that inhibits the protein kinase activities via antiangiogensis, oncogenesis, and tumor microenvironment. Regorafenib is approved as a single agent for treatment of patients with metastatic colorectal cancer (CRC), who failed previous therapies at an initial dose of 160 mg once daily on days 1-21 of each 28-day cycle [1, 2]. Previous randomized trials (CORRECT and CONCUR) have shown some efficacy of regorafenib, compared with that of placebo [3, 4]. However, various treatment-related adverse events, such as hand-foot skin reaction, fatigue, and rash, have been reported. The most common adverse events of grade 3-5 were hand-foot skin reaction (17%), fatigue (10%), diarrhoea (7%), hypertension (7%), and rash or desquamation (6%) [3, 4]. The incidence of serious adverse events was 9%.^3^

Studies in the real-world setting also showed high proportions of adverse events. There were many initial dose reductions, dose reductions during treatment, and discontinuations due to adverse events [5–7]. The selection of an appropriate regorafenib dose should require simultaneous consideration of anti-tumor effects. For reducing occurrence of adverse effects, several studies have tried to adjust the initial dose reduction [8–11]. For example, Suzuki T. et al. conducted a phase II study of regorafenib with a starting dose of 120 mg/day, and then the dosage was increased to 160 mg/day on day 15; however, the disease control rate was lower than the statistically expected rate.^10^ A randomized multicenter, open-label, phase II study (ReDOS) conducted a dose-escalation strategy (a starting dose 80 mg/day with a weekly escalation, per 40 mg increment, to 160 mg/day), which showed a comparable effect activity and a lower incidence of adverse events than general scheme.^11^ However, there is still no study focusing on personalized regorafenib dose modification.

The establishment of personalized regorafenib dose usage is imperative for clinical guidance, because it is helpful to optimize the anti-tumor effect and to enhance the life quality of patients. Multiple approaches should be explored to develop alternative strategies for identifying an optimal dose based on patients’ characteristics. Post hoc analysis of the CORRECT trial suggests regorafenib (160 mg once daily) has comparable efficacy in Japanese and non-Japanese subpopulations, but regorafenib-associated adverse events occurred more frequently in the Japanese subpopulation than in the non-Japanese one [12].

Japanese and non-Japanese groups were significantly different in body weight, with a difference of about 10 kg. Body weight is a possible factor to affect the anti-tumor affect and treatment-related adverse events for regorafenib treatment. Therefore, personalized regorafenib dose modification based on body weight is a promising research direction.

In the present study using a nationwide claims database in Japan, we hypothesized that low dose of regorafenib in CRC patients with low body weight would achieve similar survival benefit and incidences of adverse events as high dose in patients with high body weight.

## Methods

The present study was conducted according to the Strengthening the Reporting of Observational Studies in Epidemiology (STROBE) guidelines [13]. The study was approved by the Ethics Committee of the Graduate School and Faculty of Medicine, Kyoto University (approval number: R2719, November 18, 2020), which waived the requirement for an informed consent due to the anonymous nature of the data.

### Data sources

This study used the medical claims database maintained by Medical Data Vision Co, Ltd., (MDV; Tokyo, Japan). This database contains patient-level information on demographic characteristics; diagnoses coded according to the International Classification of Diseases, 10th revision (ICD-10); outcome at discharge; and prescription information, such as dose, number of days of supply, and quantity. The database comprises data of inpatient and outpatient medical care from 269 hospitals in different regions throughout Japan and covers approximately 17% of Japanese acute care hospitals. The distributions of age and sex in the source population were similar to those in the Japanese census, and several studies using this database have been reported [14–16].

### Study cohort

We selected data for CRC patients aged over 20 years who were diagnosed between June 2013 (the time when regorafenib was launched in Japan) and September 2018 by using ICD-10 codes in the database. We included the patients who received regorafenib and did not have a diagnosis of metastases but in whom the tumor at the primary site was considered unresectable. We excluded (1) regorafenib-treated patients with diseases other than CRC, such as a gastrointestinal stromal tumor, small intestine cancer, and liver cancer; (2) patients who had no body weight data; (3) patients who received an inappropriate initial dose (less than 80 mg or more than 160 mg); (4) patients who received other chemotherapy simultaneously with regorafenib; (5) patients who received repeated administration of regorafenib; and (6) patients who had less than 1 year follow-up period. We set the cutoff point as 60 kg body weight and median average dose across the whole follow-up period (120 mg). The cutoff value of 60 kg was close to the median body weight (56.7 kg) and was set for easy understanding. By using the average dose, we investigated not only the appropriate initial dose but also the appropriate maintenance dose. The high average dose group (>120 mg) includes many patients starting 160 mg initial dose and patients starting reduced initial dose of 120 mg and later increasing to 160 mg. We divided the patients into four groups based on different weight and dose combinations: heavy weight/low average dose group (heavy/low group), heavy weight/high average dose group (heavy/high group), light weight/high average dose group (light/high group), light weight/low average dose group (light/low group).

### Outcome measures

The primary outcome of this study was overall survival (OS) between heavy/high group and light/low group adjusted by inverse probability of treatment weighting (IPTW). OS was defined as the time from regorafenib initiation until death from any cause. Patients who lost to follow-up or survived until September 2018 were censored at the date of the last visit. We also retrieved the following patient information from the database: age, body weight, body mass index (BMI), comorbidities, the primary site of disease, metastatic sites, previous systemic anticancer agents administered, doctor’s department, and subsequent anticancer agents administered. We utilized data on comorbidities registered before the first prescription of regorafenib by using ICD-10 codes.

### Statistical analysis

Demographics, disease characteristics, and cancer history of heavy/high and light/low groups are displayed. Descriptive summaries of continuous data present the group mean and standard deviation. Descriptive summaries of categorical data present the category counts as frequencies and percentages. To compare the drug exposure, adverse events, and use of subsequent anticancer agents between the heavy/high and light/low groups, we used two-sample t-test or Mann-Whitney U test for continuous variables and Fisher’s exact test for categorical variables.

To account for selection bias, the observed difference in patient characteristics were adjusted by using inverse probability of treatment weighting (IPTW) method. Specifically, we first estimated the propensity score which reflecting the probability of being in heavy/high vs. light/low groups, using a logistic regression. The following patient characteristics and clinical risk factors were included in the models: age, sex, the primary site of disease, comorbidities, previous systemic anticancer agents, and doctor’s department. When comparing heavy/high group vs. heavy/low group or light/high group vs. light/low group, body weight was also included in the logistic regression as a confounding factor. The propensity scores were then used to weight each patient with the aim of balancing the characteristics between two groups (e.g., heavy/high vs. light/low). The imbalance of baseline characteristics between groups were assessed using standardized differences (SD) [17] in the original (unweighted) study population and the weighted population, respectively. A SD less than 0.1 was considered indicative of a negligible imbalance between groups.

The adjusted Kaplan–Meier curves and log-rank tests based on IPTW were computed to compare OS between different groups. Moreover, an inverse probability weighted Cox proportional hazards regression model with dose group as the sole predictor was used to determine the relative change in hazards (i.e., the IPTW-adjusted hazards ratio [HR]) associated with high dose or low dose. To check the robustness of the results in the present study, we performed sensitivity analysis by setting the median body weight (56.7 kg) of all patients as the cut-off. We further performed subgroup analysis to investigate the IPTW-adjusted HR of high vs. low dose within the light and high body weight subgroups. A nominal *P* < 0.05 (two-sided) was considered statistically significant. Statistical analyses were performed using SAS (Version 9.4; SAS Institute, Cary, NC) and R statistical software version 4.03 (https://www.r-project.org/).

## Results

### Patients

**Fig. 1:**
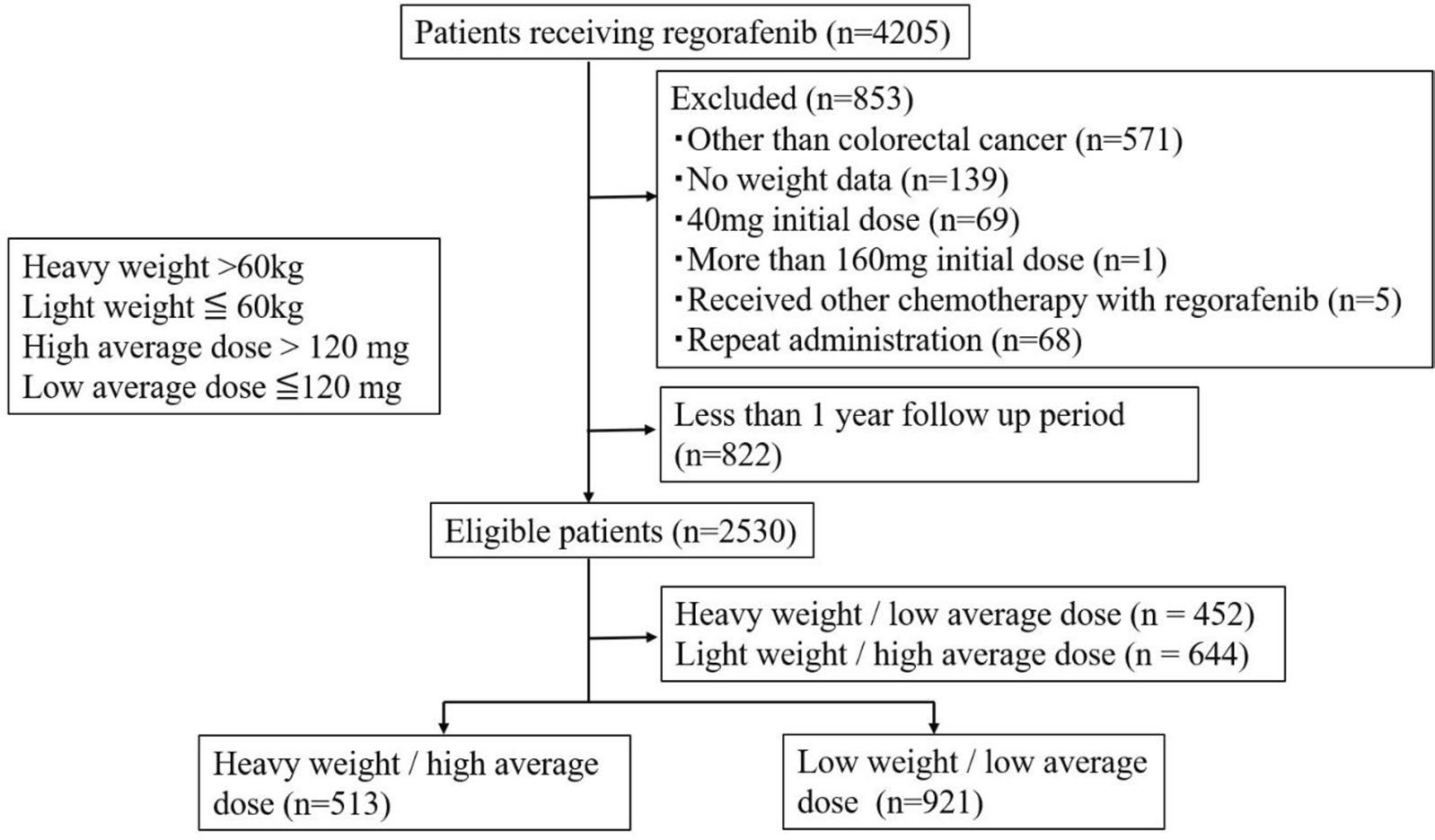
Flow diagram in the study population

The number of patients who received regorafenib was 4205, of whom we excluded 1675 patients based on the exclusion criteria. The patient number of heavy/high group was 513, light/low group was 921, heavy/low group was 579, and light/high group was 577. The flow diagram for the present study is shown in Fig 1. Table 1 displays demographics, disease characteristics, and cancer history of heavy/high and light/low groups, in addition to the comparisons of unadjusted and adjusted standardized difference between these two groups. Compared with the heavy/high group, patients in light/low group were more likely to be older, be treated by doctors from Department of Surgery, more female sex, less likely to have diabetes mellitus, to be administered systemic anticancer agents of fluorouracil, tegafur/gimeracil/oteracil, and irinotecan. After IPTW adjustment, the characteristics in the weighted population for heavy/high and light/low groups are comparable (SD for most variables was < 0.1).

**Table 1:** Patient characteristics

Characteristic	Heavy/high group (*n*=513)	Light/low group (*n*=921)	Standardized difference	Standardized difference (after IPTW adjustment)
Mean age (SD)	61.8 (10.3)	67.9 (9.6)	0.604	0.013
>65, n (%)	199 (39)	583 (62)	0.506	0.040
Sex (male), n (%)	452 (88)	411 (45)	1.037	0.106
Mean body weight (SD), kg	69.6 (7.7)	49.9 (6.8)	2.708	NA
Mean body mass index (SD), kg/m^2^	24.9 (2.9)	20.3 (2.7)	1.635	NA
missing	5	3		
≦18.5, n (%)	7 (1)	256 (28)	0.808	NA
Initial dose, n (%)			2.539	NA
160 mg	466 (91)	114 (12)		
120 mg	34 (7)	489 (53)		
80 mg	13 (3)	318 (35)		
Comorbidity, n (%)				
Hypertension	295 (58)	533 (58)	0.007	0.194
Hyperlipidemia	87 (17)	169 (18)	0.036	0.033
Diabetes mellitus	193 (38)	249 (27)	0.228	0.023
Hepatitis B	51 (10)	92 (10)	0.002	0.077
Hepatitis C	14 (3)	26 (3)	0.006	0.069
Peripheral neuropathy	178 (35)	343 (37)	0.053	0.125
Hand-foot syndrome	40 (8)	87 (9)	0.059	0.086
Primary site of disease, n (%)			0.069	0.070
Colon	273 (53)	517 (56)		
Rectum	153 (30)	247 (27)		
Colon and rectum	87 (17)	157 (17)		
Metastatic sites, n (%)				
Liver	359 (70)	618 (67)	0.062	0.031
Lung	252 (49)	485 (53)	0.071	0.050
Lymph node	127 (25)	229 (25)	0.002	0.003
Peritoneum	136 (27)	276 (30)	0.077	0.153
Bone	89 (17)	142 (15)	0.052	0.010
Brain	38 (7)	63 (7)	0.022	0.008
Others	47 (9)	127 (14)	0.146	0.012
Number of metastatic sites (≧3), n (%)	150 (29)	309 (34)	0.093	0.143
Time from diagnosis of metastases				
Mean (SD), months	26.9 (21.0)	27.6 (22.0)	0.034	0.088
≧18 months, n (%)	305 (63)	572 (65)	0.044	0.040
Missing metastatic diagnosis, n (%)	25 (5)	36 (4)		
Previous systemic anticancer agents, n (%)				
Trifluridine	141 (28)	263 (29)	0.024	0.140
Fluorouracil	352 (69)	579 (63)	0.121	0.154
Capecitabine	177 (35)	334 (36)	0.037	0.104
Tegafur/gimeracil/oteracil	145 (28)	308 (33)	0.112	0.001
Tegafur	37 (7)	62 (7)	0.019	0.074
Oxaliplatin	379 (74)	672 (73)	0.021	0.211
Irinotecan	439 (86)	741 (81)	0.137	0.169
Bevacizumab (anti-VEGFR antibody)	400 (78)	716 (78)	0.006	0.091
Cetuximab (anti-EGFR antibody)	104 (20)	159 (17)	0.077	0.023
Panitumumab (anti-EGFR antibody)	165 (32)	242 (26)	0.130	0.025
Ramucirumab (anti-VEGFR antibody)	18 (4)	28 (3)	0.026	0.018
Mean number of previous anticancer agents (SD)	4.6 (1.9)	4.5 (1.9)	0.072	0.198
Any previous targeted therapy, n (%)	455 (89)	794 (86)	0.075	0.036
Department, n (%)			0.221	0.106
Medical oncology	52 (10)	87 (9)		
Internal medicine	180 (35)	235 (26)		
Surgery	281 (55)	599 (65)		

*Abbreviations*: *EGFR* Epidermal growth factor receptor, *VEGFR* Vascular endothelial growth factor receptor, *SD* Standard deviation

### Effectiveness

**Fig. 2:**
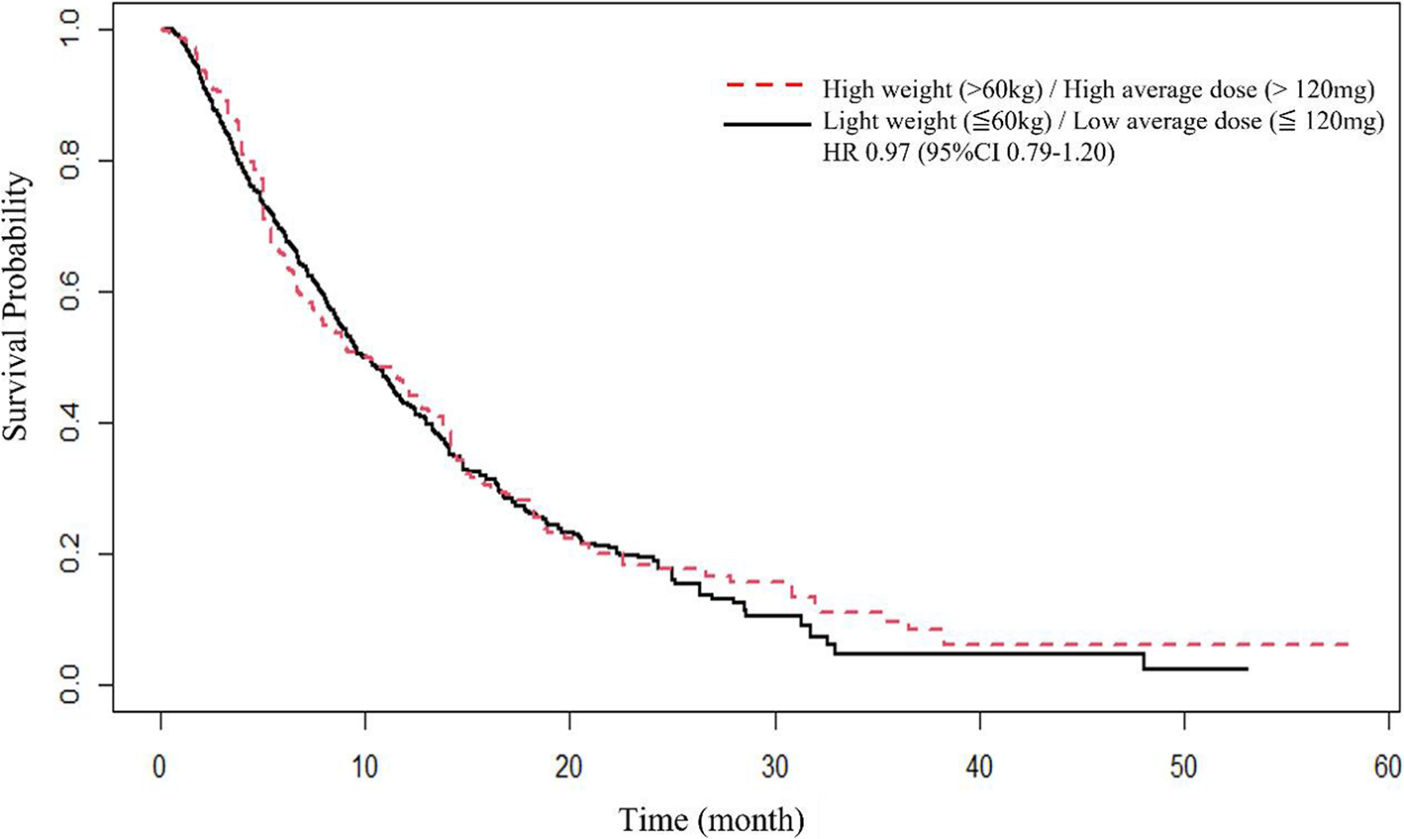
Overall survival. Kaplan-Meier estimates between the High weight (>60kg) /High average dose (>120mg) group and the Light weight (≦60kg)/Low average dose (≦120mg) group after IPTW adjustment

**Fig. 3:**
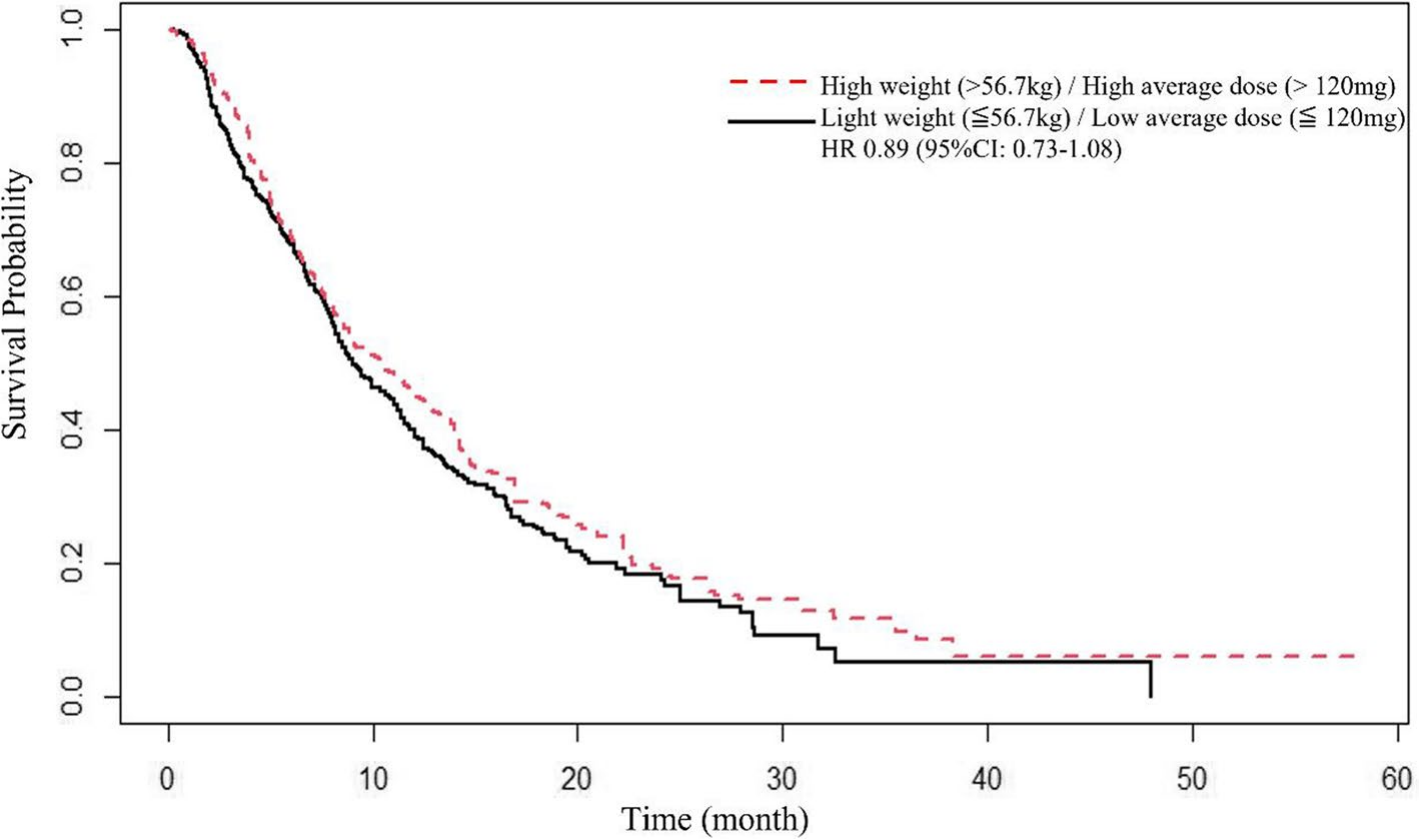
Overall survival. Kaplan-Meier estimates between the High weight (>56.7kg) /High average dose (>120mg) group and the Light weight (≦56.7kg)/Low average dose (≦120mg) group after IPTW adjustment

There was no significant difference in OS between the heavy/high group and the light/ low group after IPTW adjustment (HR=0.97; 95% CI 0.79 – 1.20, *p*=0.81)) (Fig 2). Comparison of drug exposure, adverse events, and subsequent anticancer agents between these two groups was shown in Table 2. Treatment duration was longer (51 days vs. 49 days, *p*=0.043) and total dosage was higher (5600 mg vs. 3160 mg, *p* < 0.001) in heavy/high group. There were no significant differences in the adverse events. Sensitivity analysis based on median body weight (56.7 kg) showed no significant difference in OS (IPTW adjusted HR=0.89; 95% CI 0.73 – 1.08, *p*=0.19) (Fig 3). In the subgroup of light-weight patients, higher average dose was associated with shorter OS (IPTW adjusted HR=1.21, 95% CI 1.05 – 1.39, , *p*=0.01) (Table 3) while among the heavy-weight patients, there was no significant difference in OS between high and low dose groups(IPTW adjusted HR=1.14, 95% CI 0.95 – 1.37, *p*=0.17) (Table 3). Comparison of drug exposure, adverse events, and subsequent anticancer agents in the subgroup of light-weight patients was shown in Supplemental Table 1. Mean time of treatment duration was longer in light/low group and some adverse events rates (oral mucositis, rash/desquamation, and hepatotoxicity) were higher in light/high group.

**Table 2:** Comparison of drug exposure, adverse events, and subsequent anticancer agents

	Heavy/ high group (*n*=513)	Light/low group (*n*=921)	*p* value
Mean time of treatment duration (SD), day	89 (109)	82 (106)	0.043
Median time of treatment duration (IQR), day	51 (21-106)	49 (20-103)	0.043
Median total dosage (IQR), mg	5600 (3360-11200)	3160 (1680-6160)	<0.001
Adverse events, n (%)			
Hand-foot skin reaction	161 (31)	279 (30)	0.67
Hypertension	138 (27)	216 (23)	0.15
Nausea	53 (10)	108 (11)	0.42
Diarrhea	52 (10)	69 (7)	0.084
Oral mucositis	48 (9)	76 (8)	0.48
Rash/desquamation	39 (8)	71 (8)	0.94
Fever	17 (3)	27 (3)	0.69
Hepatotoxicity	9 (2)	9 (1)	0.21
Fatigue	6 (1)	14 (2)	0.59
Subsequent anticancer agents, n (%)			
Any anticancer agents	256 (50)	399 (43)	0.017
Trifluridine/ tipiracil	182 (35)	281 (31)	0.054
Fluorouracil	62 (12)	80 (9)	0.039
Capecitabine	31 (6)	43 (5)	0.26
Tegafur/gimeracil/oteracil	53 (10)	59 (6)	0.008
Tegafur	13 (3)	18 (2)	0.47
Oxaliplatin	57 (11)	67 (7)	0.013
Irinotecan	66 (13)	85 (9)	0.032
Bevacizumab (anti-VEGF antibody)	76 (15)	105 (11)	0.062
Cetuximab (anti-EGFR antibody)	23 (5)	25 (3)	0.074
Panitumumab (anti-EGFR antibody)	30 (6)	37 (4)	0.12
Aflibercept (anti-VEGF antibody)	7 (1)	10 (1)	0.64
Ranibizumab (anti-VEGF antibody)	16 (3)	38 (4)	0.34

*Abbreviations*: *TFTD* Trifluridine/tipiracil, *IQR* Interquartile range, *EGFR* Epidermal growth factor receptor, *VEGF* Vascular endothelial growth factor receptor, *SD* Standard deviation, *IQR* Interquartile range

**Table 3:** Comparison of OS between high and low does groups among light-weight and heavy-weight patients

	Hazard ratio (95% CI)	Hazard ratio (95% CI) after IPTW adjustment
Light/high vs. light/low	1.17 (1.03 – 1.33)	1.21 (1.05 – 1.39)
Heavy/high vs. heavy/low	1.21 (1.02 – 1.44)	1.14 (0.95 – 1.37)

*Abbreviations*: *OS* Overall survival, *CI* Confidence interval

## Discussion

Using a nationwide claims database, this study firstly investigated the appropriate dose of regorafenib based on body weight to improve survival. The present study showed that light-weight CRC patients who received reduced average dose may have no significant improvement/difference in survival or adverse event as heavy-weight patients who received high average dose. High average dose use among light-weight patients was associated with shorter OS. Among the heavy-weight patients, dose reduction of regorafenib had similar efficacy as the standard dose 160 mg once daily.

In CRC patients, previous randomized trials of regorafenib demonstrated not only a good efficacy but also a high proportion of adverse events [3, 4]. Since adverse events were observed mainly within the first cycles of treatment, [4, 5] several studies have been conducted focusing on the initial dose reduction [8–11]. These studies reported that the reduced initial dose groups experienced lower treatment-related toxicity and comparable effect, compared with the 160 mg initial dose groups. However, there were few discussions of reducing the maintenance dose.

The adequate initial and maintenance dose of cytotoxic anticancer agents is determined based on the results of the Phase I study. Because these agents show a typical dose-toxicity relationship, an adequate dose would be maximum tolerated dose (MTD). On the other hand, most oral targeted anticancer agents including regorafenib have an extensive interindividual pharmacokinetic variability and a narrow therapeutic window [18]. Therefore, an initial and maintenance dose finding of new oral targeted therapies should be determined based on individual patient’s characteristics [19]. Regorafenib would be given later in the series of treatments, and given the patient’s quality of life, setting a minimum dose as the initial or maintenance dose with satisfying anti-tumor effect and less adverse events may be the most appropriate treatment strategy. However, the initial dose of regorafenib was set as MTD from a traditional Phase I trial, and it can be sub-optimal [2, 20, 21]. Ideally, the dose should be personalized based on patients’ predictive bio-markers and/or blood drug concentrations, but currently there is no definite available method [22]. In the main analysis, the median duration of treatment was within two months, and a certain proportion of patients were considered to be discontinued due to adverse events. The proportion of adverse events and treatment duration were not different between the heavy/high group and the light/low group. On the other hand, the total dosage was significantly higher in the heavy/high group. In subgroup analysis with light-weight patients, the light/low group showed a longer treatment duration. This result might be due to the tendency of the lower adverse events in the light/low group. Therefore, a reduced-dose administration for both initial dose and average dose should be done more aggressively, especially for light-weight patients.

Due to the nature of the database, the present study had some limitations. First, several pieces of information were missing (e.g., performance status, grades of adverse events, *KRAS* mutation, reasons for treatment discontinuation, and therapeutic evaluation). We could not determine progression-free survival and disease control rates from the database. Second, there were many censored cases in the OS analysis. If patients changed hospitals or decided to opt for the end-of-life care at other places such as own home or hospice, it is impossible to follow-up patient outcomes. Finally, since most of the patients were Japanese, the patients in our study were not representative of the general population in the world. In a previous Japanese study, the patients were older and thinner and had more adverse events [12]. Thus, similar studies based on population from more geographically diverse regions are needed.

## Conclusion

We firstly performed a comparative analysis of heavy-weight CRC patients treated with high average regorafenib dose and light-weight CRC patients treated with low average regorafenib dose using a large nationwide cohort of patients with CRC. There was no significant difference in survival, and reduced doses of regorafenib were considered appropriate for light-weight patients. Moreover, heavy-weight CRC patients may achieve similar survival benefit with reduced doses as compared to the standard dose. The results of this study are helpful in providing appropriate guidance about regorafenib dose to CRC patients.

### Supplementary Information

**Additional file 1: Supplemental Table 1.** Comparison of drug exposure, adverse events, and subsequent anticancer agents.

## Data Availability

The data that support the findings of this study are available from Medical Data Vision Co, Ltd. But restrictions apply to the availability of these data, which were used under license for the current study, and so are not publicly available. Data are however available from the corresponding authors upon reasonable request and with permission of Medical Data Vision Co, Ltd.

## Abbreviations

BMI: Body mass index
CRC: Colorectal cancer
IPTW: Inverse probability of treatment weighting
MTD: Maximum tolerated dose
OS: Overall survival
SD: Standardized differences
