# Supplementary material for: Appropriate dose of regorafenib based on body weight of colorectal cancer patients: a retrospective cohort study
Source: BMC Cancer. 2023 Dec 21;23:1268. doi: 10.1186/s12885-023-11720-6 (PMC10740272; doi:10.1186/s12885-023-11720-6)
Supplement: Supplementary file 1 — Additional file 1: Supplemental Table 1. Comparison of drug exposure, adverse events, and subsequent anticancer agents. [file 12885_2023_11720_MOESM1_ESM.docx]

Supplemental Table 1 Comparison of drug exposure, adverse events, and subsequent anticancer agents

|  | Light/ low group  (n=921) | Light/ high group (n=644) | p value |
| --- | --- | --- | --- |
| Mean time of treatment duration (SD), day | 82 (106) | 67 (91) | <0.001 |
| Median time of treatment duration (IQR), day | 49 (20-103) | 36 (14-77) | <0.001 |
| Mean total dosage (SD), mg | 5142 (5998) | 6628 (7701) | <0.01 |
| Median total dosage (IQR), mg | 3160 (1680-6160) | 3500 (2240-7280) | <0.001 |
| Adverse events, n (%) |  |  |  |
| Any adverse event | 542 (59) | 396 (61) | 0.29 |
| Hand-foot skin reaction | 279 (30) | 196 (30) | 0.95 |
| Hypertension | 216 (23) | 136 (21) | 0.11 |
| Nausea | 108 (11) | 73 (11) | 0.81 |
| Diarrhea | 69 (7) | 65 (10) | 0.070 |
| Oral mucositis | 76 (8) | 80 (12) | 0.007 |
| Rash/desquamation | 71 (8) | 70 (11) | 0.032 |
| Fever | 27 (3) | 17 (3) | 0.73 |
| Hepatotoxicity | 9 (1) | 15 (2) | 0.032 |
| Fatigue | 14 (2) | 4 (1) | 0.10 |
| Subsequent anticancer agents, n (%) |  |  |  |
| Any anticancer agents | 399 (43) | 269 (42) | 0.54 |
| Trifluridine/ tipiracil | 281 (31) | 192 (30) | 0.77 |
| Fluorouracil | 80 (9) | 66 (10) | 0.30 |
| Capecitabine | 43 (5) | 30 (5) | 0.99 |
| Tegafur/gimeracil/oteracil | 59 (6) | 47 (7) | 0.49 |
| Tegafur | 18 (2) | 8 (1) | 0.28 |
| Oxaliplatin | 67 (7) | 44 (7) | 0.74 |
| Irinotecan | 85 (9) | 72 (11) | 0.21 |
| Bevacizumab (anti-VEGF antibody) | 105 (11) | 71 (11) | 0.82 |
| Cetuximab (anti-EGFR antibody) | 25 (3) | 29 (5) | 0.056 |
| Panitumumab (anti-EGFR antibody) | 37 (4) | 26 (4) | 0.98 |
| Aflibercept (anti-VEGF antibody) | 10 (1) | 9 (1) | 0.58 |
| Ranibizumab (anti-VEGF antibody) | 38 (4) | 26 (4) | 0.93 |

TFTD: trifluridine/tipiracil, IQR: interquartile range, EGFR: epidermal growth factor receptor, VEGF: vascular endothelial growth factor receptor, SD: standard deviation, IQR: interquartile range
